# Overexpression of NCAPG in ovarian cancer is associated with ovarian cancer proliferation and apoptosis via p38 MAPK signaling pathway

**DOI:** 10.1186/s13048-022-01030-z

**Published:** 2022-08-19

**Authors:** Haiting Yu, Dan Zou, Na Ni, Suxian Zhang, Qin Zhang, Lihua Yang

**Affiliations:** Department of Obstetrics and Gynecology, The Second Affiliated Hospital of Kunming Medical University, 374 Dianmian Blvd, Wuhua, Kunming, Yunnan 650101 P.R. China

**Keywords:** NCAPG, shRNA, p38MAPK, Ovarian cancer, Cell cycle

## Abstract

**Background:**

Non-SMC condensin I complex subunit G (NCAPG), a member of the subunit of condensin complex, is significantly overexpressed in various cancers and involved in the pathogenesis of cancers. However, the roles of NCAPG in ovarian cancer remain unclear.

**Methods:**

The mRNA expression, overall survival, and disease-free survival of NCAPG in ovarian cancer were analyzed by GEPIA and KM plotter database, and the expression levels of NCAPG in OC tissues and cell lines were determined by qPCR and immunohistochemistry analysis. shRNA targeting NCAPG gene (sh-NCAPG) was utilized to knock down NCAPG expression in OVCAR3 and SKOV3 cells. Subsequently, CCK-8 assay, colony formation assay, transwell invasion assay and flow cytometric analysis were performed to detect the effect of NCAPG on OC cell proliferation, apoptosis, and invasion. Finally, western blot assays were performed to detect the mechanism of NCAPG in ovarian cancer.

**Results:**

Analysis using GEPIA and KM plotter database showed NCAPG was upregulated in ovarian cancer and negatively associated with the survival of OC patients. qPCR and immunohistochemistry analysis confirmed it was highly expressed in both ovarian cancer tissues and cells. The silencing of NCAPG inhibited OC cell proliferation and invasion, and induced cell apoptosis. Additionally, flow cytometric analysis revealed that NCAPG knockdown arrested the cell cycle at G2 and S phases. Furthermore, we also found that downregulation of NCAPG could suppress OC cell proliferation and invasion via activating the p38 MAPK signaling pathway.

**Conclusion:**

Our results suggest that NCAPG exhibits an important role in the development and progression of ovarian cancer and implicates NCAPG as a potential therapeutic target in ovarian cancer.

## Background

Ovarian cancer (OC) is the most lethal gynecologic disease that ranks second in incidence and first in mortality, with five-year survival rates below 45% [1, 2]. The high mortality of ovarian cancer is due to the lack of specific symptoms in the early stage and the absence of effective screening and therapeutic strategies [3]. A broad scope of genetic and epigenetic modifications plays an essential role in the development and tumorigenesis of ovarian cancers. Thus, further exploration of the epigenetic modifications related genes of OC will offer valuable insights for developing therapy targets and effective predicting prognosis.

Non-SMC condensin I complex subunit G (NCAPG), a mitosis and meiosis associated chromosomal condensing protein [4], is involved in mitotic chromosome condensation and is related to DNA methylation [5, 6]. NCAPG is a member of non-SMC subunits in condensing I, which is involved in tumor cell survival, proliferation, migration, and metastasis [7]. NCAPG may promote the occurrence and development of tumors by regulating the cell cycle, cell aging, and mismatch repair [8]. NCAPG deficiency increases apoptosis and inhibits tumor cell proliferation [9]. Previous studies have shown that NCAPG is highly expressed in many malignant tumors, such as gastric cancer, liver cancer, glioma, and prostate cancer, and its expression level is closely related to the poor prognosis of the tumors [10–12]. However, there is remain unknown the effects of NCAPG in ovarian cancer.

The P38MAPK signaling is a critical pathway for OC development and participates in various biological characteristics, including tumor development, drug resistance, and metastasis regulation [13, 14]. Studies published in the last decade showed a dual role for the p38 MAPK signaling pathway in cancer development as it displays both tumorigenic and tumor-suppressive effects [15]. P38MAPK signaling pathway acts as a tumor suppressor linked with the stimulation of p53-dependent cell cycle arrest [16]. Furthermore, activation of the p38MAPK/p53 pathway plays a key role in arresting cell cycle and promoting apoptosis [17]. Feng et al. showed that silencing Wip1 promoted SKOV3 cell apoptosis by activating the p38MAPK/p53 signaling pathway [18]. Chen et al. found that Fe2 + can inhibit granulosa cell proliferation and arrest the cell cycle by activating the ROS-mediated p38MAPK/p53 signaling pathway [19]. However, whether p38MAPK/p53 signaling is involved in the role of NCAPG in OC remains to be further investigated. Therefore, we carry out experiments to figure out the effects of NCAPG on OC cell progression via the p38 MAPK/P53 signaling pathway.

## Methods

### Expression and survival analysis

The GEPIA database is an online tumor database integrated with RNA sequencing expressions of 9,736 tumor samples and 8,587 normal samples from the Cancer Genome Atlas (TCGA) and the Genotype-Tissue Expression (GTEx) databases [20]. The expression of NCAPG in OC and normal tissues were analyzed in and visually displayed in boxplot format in the GEPIA database (http://gepia.cancer-pku.cn/), and log2 (TPM + 1) was used for log-scale.

Kaplan Meier-plotter (KM plotter) is another cancer database, but different from the GEPIA database, which is based on TCGA, EGA, and GEO (Affymetrix microarrays only) database [21]. The overall survival (OS) and progression-free survival (PFS) of the NCAPG-low and NCAPG-high expression subgroups were compared in the KM plotter (http://kmplot.com/analysis/). The hazard ratio (HR) with the 95% confidence interval and the log-rank P-value were calculated and displayed on the plot, and the number-at-risk is displayed below the curves. Log-rank *P*-value < 0.05 was considered statistically significant.

### Tissue microarray

A tissue microarray harboring seventy ovarian cancer tissues and ten para-carcinoma tissues was purchased from bioaitech(Xian, China). Seventy cases of ovarian cancer and ten cases of para-carcinoma tissues were randomly obtained from the national tertiary hospital (China) between 2010 and 2018. According to WHO histology grading: there were 8 cases in grade I, 2 cases in grade I ~ II, 2 cases in grade II, 2 cases in grade II ~ III, 54 cases in grade III, and 2 cases of no grading. Patients with preoperative radiotherapy or chemotherapy were excluded. OC and para-carcinoma tissues were collected and stored at –80 °C until analysis. Informed consent was obtained from patients before specimen collection. The study protocol was approved by the Ethics Committee of Tongxu People's Hospital of Henan Province and all investigations were conducted according to the Declaration of Helsinki.

### Immunohistochemistry

Immunohistochemistry was utilized to explore the expression patterns of NCAPG in 70 OC tissues and 10 para-carcinoma tissues. The tissue microarray was dried with drying apparatus AKT-7 (Atobo, Hubei, China), and then dewaxed with xylene (MXB biotechnologies, Fuzhou, China) and hydrating in gradient alcohol (MXB biotechnologies, Fuzhou, China), 3% hydrogen peroxide (MXB biotechnologies, Fuzhou, China) was used to inhibit endogenous peroxidase. The sections were incubated with primary antibody solution against NCAPG (1:20, abclonal, Tokyo, Japan) for 2 h. After washing three times, they were covered with HRP anti-rabbit immunoglobulin G (IgG; MXB biotechnologies, Fuzhou, China) for 30 min. After DAB(MXB biotechnologies, Fuzhou, China) staining and hematoxylin(MXB biotechnologies, Fuzhou, China)counterstaining, the sections were evaluated by two independent pathologists according to staining area and intensity. The scoring criteria for staining intensity were regarded as 0, no staining; 1, light yellow staining; 2, light brown staining; and 3, dark brown staining. The percentage of stained cells: 1, 0–25%; 2, 26–50%; 3, 51–75% and 4, 76–100%. The product of the two values was defined as the NCAPG staining scores.

### Cell culture

Human ovarian cancer cell lines SKOV3 and OVCAR3 were obtained from Kunming Institute of Zoology, Chinese Academy of Sciences (Kunming, China), and iCell Bioscience (Shanghai, China), respectively. The human ovarian epithelial cell line IOSE-80 was bought from iCell Bioscience (Shanghai, China). All cell lines were cultured in RPMI-1640 medium (Hyclon, Logan, Utah, USA) supplemented with 10% FBS (Gibco; Thermo Fisher Scientific, Inc., Waltham, MA, USA), 1% antibiotic/antimycotic solution (Hyclon, Logan, Utah, USA) and placed in a 5% CO_2_ incubator at 37 °C.

### Quantitative real-time polymerase chain reaction (qRT-PCR)

Total RNA was extracted from cultured cells with Trizol reagent (Vazyme, Nanjing, China) and then reverse-transcribed to complementary DNA (cDNA) using a PrimeScriptTM RT Reagent kit (Vazyme, Nanjing, China). qRT-PCR was performed with a ABI7300 real-time quantitative PCR instrument (Applied biosystems, Foster City, CA, USA) using SYBR Green Master Mix as described by the manufacturer's protocol (TaKaRa, Dalian, China). The primers were synthesized by General (Shanghai, China) and the primer sequences used were as follows: NCAPG, forward, 5′- CGCTTTCACGACTTCAGGAT-3’ and reverse, 5′-ATAACACTGCCCGTCTAACTTCT-3′; β-actin-forward,5ʹ-CGTGCGTGACATCAAAGAGAAG -3ʹ and reverse, 5ʹ- CCAAGAAGGAAGGCTGGAAAA -3ʹ. β-actin was used as an internal control. The results were analyzed using the 2-ΔΔCt method. All RT reactions were performed in triplicate.

### Transfection of SKOV3 and OVCAR3 cells with NCAPG-shRNA plasmid

SKOV3 and OVCAR3 cells were cultured in 24-well plates to a confluence of 60–70% and then transfected with shRNAs targeting human NCAPG using Lipofectamine 3000 (Invitrogen, Carlsbad, CA, USA) following the manufacturer's protocol. Three different shRNAs were used to silence NCAPG: shNCAPG-1, shNCAPG-2, and shNCAPG-3. The NCAPG or negative control (NC) plasmid, were both purchased from GeneChem (Shanghai, China). After 48 h of transfection, the knockdown efficiency of the shRNAs was confirmed by qRT-PCR.

### Cell viability assay

The Cell Counting Kit-8 (CCK-8) assay was performed to investigate the influence of NCAPG knockdown on SKOV3 and OVCAR3 viability. In brief, after culturing and transfecting SKOV3 and OVCAR3 cells as mentioned ahead, 5 × 10^3^ cells were seeded per well in a 96-well plate. CCK8 reagent was added at 0, 24, 48, 72, 96, and 120 h, and cell viability was read with a plate reader (ELX800, BiTek, USA) at 490 nm. Three replicates of the experiments were performed.

### Colony formation assay

Colony formation assays were performed to evaluate the effect of NCAPG knockdown on OC cell anchorage-independent growth. Briefly, the transfected cells were seeded into 6-well plates at a density of 500 cells/well and cultured for 2 weeks. The culture medium was replaced every 3 days. The colonies were fixed with 4% paraformaldehyde (Solarbio, Beijing, China) and stained with 1% crystal violet (Solarbio, Beijing, China). The number of colonies was counted under a microscope (Olympus CX-23; Olympus Corporation**,** Tokyo, Japan).

### Cell invasion assays

The invasive abilities were evaluated using 8-μm transwell inserts with Matrigel precoating (BD Biosciences, San Jose, CA, USA), Briefly, the transfected cells (1 × 10^4^ cells/well) were suspended in a serum-free medium and placed in the upper chamber, culture medium containing 10% FBS was added to the bottom chamber. Following 24 h incubation, the cells that had invaded through the membrane to the lower surface were fixed and stained. Penetrated cells in three randomly selected fields were counted under an inverted microscope (Olympus CX-23; Olympus Corporation, Tokyo, Japan).

### Apoptosis and cell cycle analysis

When transfected SKOV3 and OVCAR3 cells grew to 80% confluence, the cells in suspension were collected, and the cells on the dish were digested into single-cell suspension by trypsin (Hyclon, Logan, Utah, USA). All cells were collected and mixed with 5 μL Annexin V-FITC and 5 μL propidium iodide (PI) solution (KeyGEN, Nanjing, China) for 15 min at room temperature in the dark. Apoptosis rates were determined by a BD flow cytometer (Franklin Lakes, NJ, USA). B3 quadrant represented viable cells and B2 and B4 quadrants represented apoptotic cells. For detection of cell cycle distribution, the transfected cells were digested, washed, and incubated with 1 μl RedNucleus I (Sysmex Corporation, Kobe, Japan) at 37 °C for 10 min and then measured the cell cycle distribution with a BD flow cytometer (Franklin Lakes, NJ, USA).

### Western blot analysis

The transfected cells were collected and lysed in RIPA buffer (Solarbio, Beijing, China) mixed with 1% protease inhibitor PMSF (Solarbio, Beijing, China) and incubated on ice for 30 min. The lysates were centrifuged at 14,000 g for 10 min at 4 °C, and the supernatant was collected. The extracted proteins were separated by 10% SDS-PAGE (Solarbio, Beijing, China) and transferred onto PVDF membranes (Solarbio, Beijing, China). After blocking in 5% BSA, the membranes were incubated with primary antibodies against NCAPG(1:2000, abclonal, Tokyo, Japan), P38MAPK(1:2000, proteintech, Chicago, USA), P53 (1:2000, Wanleibio, Shenyang, China), cyclinD1(1:2000, Cell Signaling Technology, Massachusetts, the United States) and β-actin (1:4000, proteintech, Chicago, USA) at 4 °C overnight. After washing with PBST the following day, the membranes were incubated with secondary antibodies (1:5000, proteintech, Chicago, USA) at room temperature for 1 h. Finally, the blots were detected by enhanced chemiluminescence (Millipore, Massachusetts, USA). Quantification of the bands was measured with ImageJ (V 1.80).

### Statistical analysis

Data were presented as mean ± standard deviation (SD) of at least three independent experiments and analyzed with GraphPad Prism 7 software (GraphPad, San Diego, CA, USA). Kaplan–Meier survival curves and the log-rank tests were used to assess significant differences. Differences between groups were analyzed by Student’s t-test for two groups and one-way ANOVA for more than two groups. *P* values < 0.05 were considered statistically significant.

## Results

### NCAPG expression and survival analysis of ovarian cancer

**Fig. 1:**
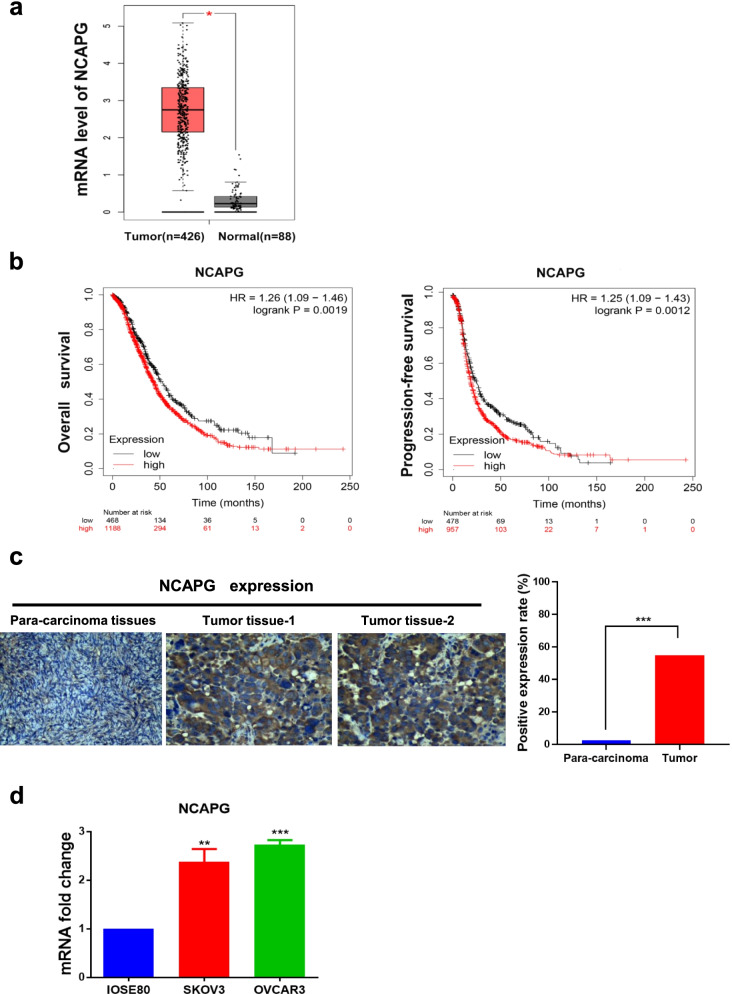
NCAPG expression in OC tissues and cell lines. **a** NCAPG mRNA expression level is determined by the GEPIA web tool. The boxplot analysis showed the expression level by log2 (TPM + 1) on a log scale. **b** KM plotter dataset was searched for the overall survival (OS, HR = 1.23, 95% CI 1.06–1.41, *P* = 0.0047) and progression-free survival (PFS, HR = 1.25, 95% CI 1.09–1.43, *P* = 0.0012). **c** Immunohistochemical staining of NCAPG protein expression in ovarian cancer tissue and para-carcinoma tissues (magnification, × 100 and × 400). **d** Quantitative real-time polymerase chain reaction of NCAPG mRNA level in two OC cell lines (SKOV3 and OVCAR3) and human ovarian epithelial cell line (IOSE80). *: *P* < 0.05, **: *P* < 0.01 and ***: < 0.001 vs control

To evaluate the relationship between NCAPG and OC, we first examined the expression of NCAPG throughout the GEPIA web tool. NCAPG mRNA expression increased significantly in ovarian cancer (Fig. 1a). Furthermore, Kaplan–Meier curves showed that the rate of 5-year OS and PFS was lower in the patients with NCAPG-high expression group than in those in the NCAPG-low expression group (Fig. 1b). Based on the bioinformatics results, we believe that NCAPG may be an essential oncogene in OC, thus exploring its role in OC is necessary.

### Upregulation of NCAPG was validated in OC tissues and cell lines

We determined the expression of NCAPG in OC cells and tissues. IHC analysis of 70 OC tissues and 10 para-carcinoma tissues showed NCAPG predominantly resides in the cytoplasm, positive staining is brown, and the intensity of NCAPG is higher in cancerous tissues than that in para-carcinoma tissues (Fig. 1c, Table 1). In addition, we observed that the expressions of NCAPG in ovarian cancer patients showed a significantly relative relationship with the histological type, FIGO stage, lymph node metastasis, and tumor grade (Table 2). Moreover, qRT-PCR analysis of two ovarian cancer cell lines (SKOV3 and OVCAR3) and human ovarian epithelial cell line IOSE80 showed that NCAPG mRNA increased in the OC cells (Fig. 1d). Our results indicated that levels of NCAPG were upregulated in OC tissues and cells, consistent with the results from the bioinformatics analysis.

**Table 1:** Protein levels of NCAPG between epithelial ovarian cancer and para-carcinoma tissues

Type of tissue	n	NCAPG		χ^2^	*P* value
		Positive (%)	Negative (%)		
Epithelial ovarian cancer	70	44 (62.86)	26 (37.14)	4.940	0.026
Para-carcinoma tissues	10	2 (20.00)	8 (80.00)

**Table 2:** Correlation between NCAPG expression and clinicopathological features in OC

Variable	n	NCAPG expression		χ^2^	*P* value
		Positive(%)	Negative (%)		
Age	70			0.53	0.467
< 50	35	13 (37.14)	22 (62.86)		
> 50	35	16 (45.71)	19 (54.29		
Histological type	70			8.754	0.003
Serous adenocarcinoma	58	32 (55.17)	26 (44.83)		
Other adenocarcinomas	12	1 (8.33)	11 (91.67)		
FIGO	70			25.426	< 0.001
I + II	57	14 (24.56)	43 (75.43)		
III + IV	13	13 (100.00)	0 (0.00)		
Lymph node metastasis	70			20.042	< 0.001
N0	58	15 (25.86)	43 (74.14)		
N1 + N2 + N3	12	12 (100.00)	0 (0.00)		
Grade	70			4.759	0.029
G1 + G2	14	2 (14.29)	12 (85.71)		
G3	54	25 (46.30)	29 (53.70)		

### NCAPG knockdown inhibits OC cell growth, proliferation, and invasion

**Fig. 2:**
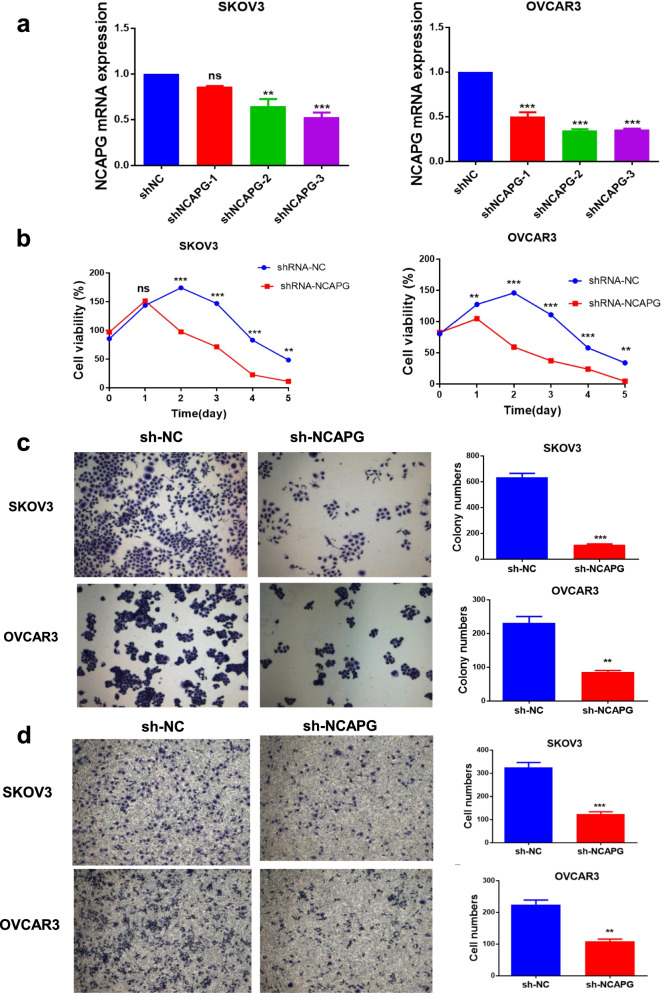
NCAPG knockdown reduced cell growth, proliferation, and invasion abilities of SKOV3 and OVCAR3 cells. **a** qRT-PCR was used to verify the effect of NCAPG knockdown with shRNAs and the shNCAPG-3 groups were employed for further experimentation. **b** Cell viability was examined by CCK8 assay on days 1, 2, 3, 4, and 5. **c** Colony formation capacity was detected by colony formation assay. **d** Cell invasion ability was assessed by transwell assay. Data are expressed as the mean ± SD for three independent experiments. *: *P* < 0.05, **: *P* < 0.01 and ***: < 0.001 vs sh-NC group

To perform the function of NCAPG in ovarian cancer, we used shRNA plasmid to knock down NCAPG in two ovarian cancer cell lines, SKOV3 and OVCAR3. As shown in Fig. 2a, The shNCAPG-3 groups successfully inhibited NCAPG expression in these cell lines and were employed for further experimentation. We measured cell viabilities for 5 consecutive days using the CCK-8 assay. The results showed that the viable cell numbers in the sh-NCAPG group were lower than those in the sh-NC group on days 2, 3, 4, and 5(Fig. 2b). Colony formation assay showed that colony formation abilities of the cells that were transfected with sh-NCAPG were reduced compared with that of sh-NC transfected cells (Fig. 2c). Consistently, the knockdown of NCAPG led to reduced invasiveness in transwell assays compared with the sh-NC groups (Fig. 2d)**.** Collectively, these data indicated that NCAPG functions as an oncogene in human OC cell lines because the knockdown of NCAPG inhibits cell growth, proliferation and invasion abilities.

### NCAPG knockdown induces OC cells apoptosis and cell cycle arrest

**Fig. 3:**
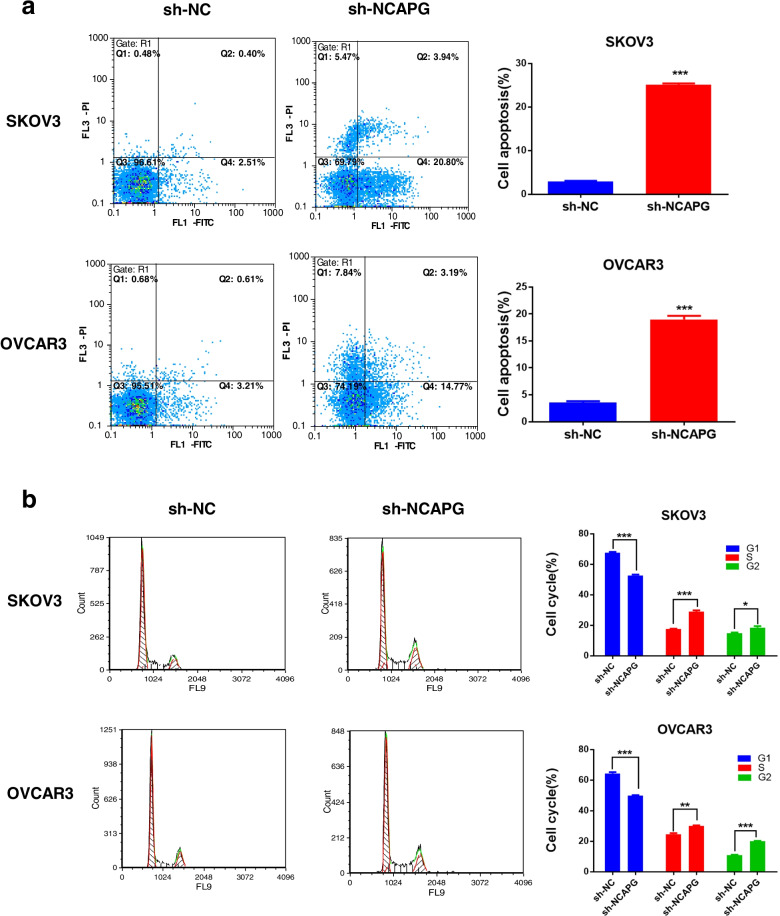
NCAPG knockdown promoted apoptosis and arrested the cell cycle of SKOV3 and OVCAR3 cells. **a** Cell apoptosis rate of transfected cells was determined by Annexin V/PI staining assay. **b** The transfected cells were subjected to flow cytometric analysis for assessment of cell cycle progression. Data are expressed as the mean ± SD for three independent experiments. *: *P* < 0.05, **: *P* < 0.01 and ***: < 0.001 vs sh-NC group

To explore the role of NCAPG in OC cell apoptosis, the apoptosis rate was analyzed by flow cytometry. A significant increase in the apoptosis rate was observed in the NCAPG shRNA group in both cell lines (Fig. 3a). NCAPG is a mitotic-associated chromosomal condensing protein responsible for the mitotic cell cycle. Hence, we detected cell cycle distribution in NCAPG knockdown cells and observed a markedly increased in the cell percentages of S and G2 phases while the cell percentages of G1 phases decreased (Fig. 3b). These results indicated that downregulation of NCAPG contributes to cell apoptosis and the cell cycle by G2 and S arrest in ovarian cancer cells.

### NCAPG knockdown promotes p38 MAPK signaling pathway activation.

**Fig. 4:**
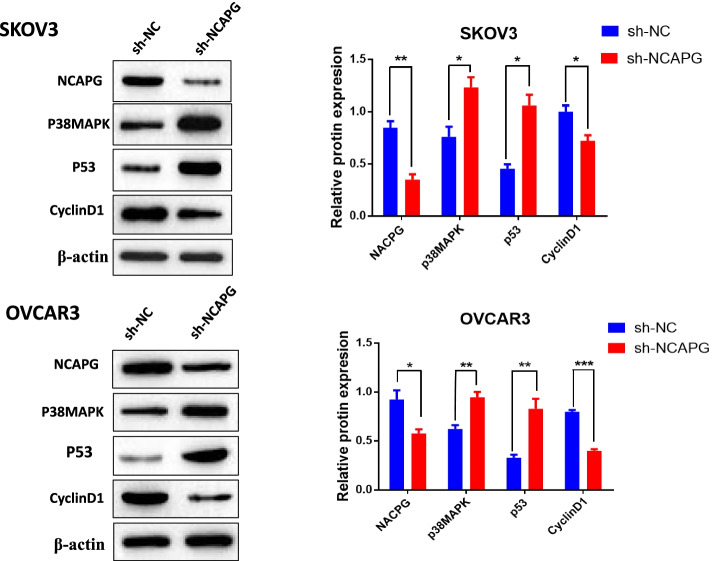
NCAPG knockdown activates the p38 MAPK signaling pathway in OC cells. Protein levels of NCAPG, p38MAPK, p53, and cyclinD1 were quantified by Western blotting. Data are expressed as the mean ± SD for three independent experiments. *: *P* < 0.05, **: *P* < 0.01 and ***: < 0.001 vs sh-NC group

We then sought to explore the molecular mechanisms through which NCAPG knockdown constrains the malignancy of ovarian cancer. we measured the p38MAPK, p53, and cyclinD1 protein expression in response to NCAPG knockdown in OC cells, and the results showed that shRNA-mediated knockdown of NCAPG increased the levels of p38MAPK and p53 and reduced the levels of cyclin D1 in both SKOV3 and OVCAR3 cells (Fig. 4a-b). Thus, these data showed that knockdown of NCAPG by shRNA interference promoted the activation of the p38MAPK /p53 signaling pathway in OC cells.

## Discussion

NCAPG, a mitotic associated chromosomal condensing protein located on human chromosome 4p15.32, is encoded by the gene NY-MEL-3 [22], which is also involved in global DNA methylation and gene-specific DNA methylation [23]. Current studies have demonstrated that NCAPG was involved in the pathogenesis of a variety of malignant tumors, such as hepatocellular carcinoma, renal cell carcinoma, breast cancer, and prostate cancer [24–27]. However, no study has clarified the role of NCAPG in ovarian cancer. According to the GEPIA dataset (http://gepia.cancer-pku.cn/), an intensive expression of NCAPG was found in OC. In addition, the Kaplan–Meier Plotter database (http://kmplot.com/analysis/) was applied to analyze the relationship between the NCAPG expression levels and the prognosis of patients with ovarian cancer. The results showed that higher expression predicted a worse prognosis for patients with ovarian cancer. In the present study, we assessed tumor specimens derived from ovarian cancer patients for NCAPG protein expression using immunohistochemistry. Subsequently, the experiment results indicated the upregulation of NCAPG in OC tissues. It was identical to those reported in the dataset. Moreover, we also confirmed the upregulation of NCAPG mRNA expression in two OC cell lines compared with ovarian epithelial cell line IOSE-80 determined by qPCR. To confirm the function of NCAPG in OC, we knocked down NCAPG expression in ovarian cancer OVCAR3 and SKOV3 cells with shRNA plasmid vectors. We found that downregulation of NCAPG in OC cells inhibited cell viability and colony efficiency as well as cell invasion ability. Furthermore, flow cytometric analysis showed that NCAPG knockdown arrested the cell cycle in G2 and S phases, and promoted cell apoptosis. Together, the above-mentioned observations indicated that NCAPG functions as an oncogene in the development of OC. Therefore, the related mechanisms of NCAPG deserve further investigation.

The p38MAPK, a subfamily member of MAPKs (Mitogen-activated protein kinases), is a typical stress-activated kinase that mediates apoptosis signaling [28]. Dysregulation of the p38MAPK signaling has been considered a key regulator of cell growth, proliferation, and cell cycle progression in many cancers [29–31]. In addition, these aforementioned responses participated in both apoptosis and cell growth depending on the cell type or the applied stimulus. In vitro and in vivo research demonstrated that LicA induced mitochondria-mediated intrinsic apoptosis through the activation of the p38MAPK pathway in human osteosarcoma cells [32]. Other evidence reveals that the activation of phosphorylated p38MAPK expression mediates TNF‑α‑induced apoptosis in glioma cells [33]. While the role of p38MAPK is controversial in several tumor cells, activation of the p38MAPK pathway in response to transcription factor p53 occurs in ovarian cancer. P53, identified as a tumor suppressor gene that integrates numerous signals controlling cell cycle arrest as well as cell apoptosis, is a critical propagator of P38 MAPK signaling [34, 35]. For example, Lee et al.confirmed that Orostachys japonicus induces p53- dependent cell cycle arrest and activates the p38 MAPK pathway, thus leading to apoptosis in ovarian cancer [36]. Feng et al.have demonstrated that silencing proto-oncogene Wip1 (Wild-type p53-induced phosphatase 1) promotes SKOV3 cell apoptosis through the activation of the p38 MAPK signaling pathway [18]. The present results echo prior findings that silencing NCAPG significantly activation of the p38 MAPK/p53 signaling pathway. In SKOV3 and OVCAR3 cells, downregulation of NCAPG and examined the activity of p38 MAPK and its target genes, including p53 and cyclin D1. Western blot analysis indicated that p38 MAPK and p53 expression were increased and cyclin D1 expression was decreased after cells were transfected with shRNA interference vectors targeting the NCAPG gene. Taken together, it was revealed that p38 MAPK/p53/cyclinD1 may be a critical mechanism of NCAPG-mediated tumorigenesis in ovarian cancer. However, future investigations are warranted to understand the roles of p38 MAPK in the effects of NCAPG knockdown on cell cycle arrest and apoptosis induction in OC cells.

## Conclusions

In summary, our results demonstrated for the first time the overexpression of NCAPG in ovarian cancer. NCAPG knockdown significantly inhibited cell proliferation, caused cell-cycle arrest, induced cell apoptosis, and suppressed the abilities of cell invasion. Notably, we illuminated that NCAPG knockdown may inhibit the development of ovarian cancer via the p38 MAPK signaling pathway, which suggests that NCAPG may be a prognostic therapeutic target in ovarian cancer.

## Data Availability

All data generated or analyzed during this study are included in this published article.

## Abbreviations

NCAPG: Non-SMC condensin I complex subunit G
GEPIA: Gene Expression Profiling Interactive Analysis
KM: Kaplan–Meier
qRT-PCR: Quantitative real-time polymerase chain reaction
OC: Ovarian cancer
CCK-8: Cell counting kit 8
ShRNA: Short hairpin RNA
p38 MAPK: P38 mitogen-activated protein kinase
SMC: Structural maintenance of chromosomes
Wip1: Wild-type p53-induced phosphatase 1
ROS: Reactive oxygen species
TCGA: The Cancer Genome Atlas
GTEx: Genotype-Tissue Expression
EGA: European Genome-phenome Archive
GEO: Gene Expression Omnibus
OS: Overall survival
PFS: Progression-free survival
HR: Hazard ratio
RPMI: Roswell Park Memorial Institute
FBS: Fetal bovine serum
cDNA: Complementary DNA
NC: Negative control
RIPA: Radio Immunoprecipitation Assay
PMSF: Phenylmethanesulfonyl fluoride
SDS-PAGE: Sodium dodecyl sulfate polyacrylamide gel electrophoresis
PVDF: Polyvinylidene fuoride
BSA: Bovine serum albumin
GAPDH: Glyceraldehyde-3-phosphate dehydrogenase
PBST: Phosphate buffered saline and twen-20
SD: Standard deviation; mRNA: Messenger RNA
CI: Confidence interval
MAPKs: Mitogen-activated protein kinases
LicA: Licochalcone A
TNF: Tumor necrosis factor

## References

[1] Bray F, Ferlay J, Soerjomataram I, Siegel RL, Torre LA, Jemal A (2018). Global cancer statistics 2018: GLOBOCAN estimates of incidence and mortality worldwide for 36 cancers in 185 countries. CA Cancer J Clin.

[2] Momenimovahed Z, Tiznobaik A, Taheri S, Salehiniya H (2019). Ovarian cancer in the world: epidemiology and risk factors. Int J Women's Health.

[3] Dong X, Men X, Zhang W, Lei P (2014). Advances in tumor markers of ovarian cancer for early diagnosis. Indian J Cancer.

[4] Eberlein A, Takasuga A, Setoguchi K, Pfuhl R, Flisikowski K, Fries R (2009). Dissection of genetic factors modulating fetal growth in cattle indicates a substantial role of the non-SMC condensin I complex, subunit G (NCAPG) gene. Genetics.

[5] Hudson DF, Marshall KM, Earnshaw WC (2009). Condensin: Architect of mitotic chromosomes. Chromosome Res.

[6] Legagneux V, Cubizolles F, Watrin E (2004). Multiple roles of Condensins: a complex story. Biol Cell.

[7] Ai J, Gong C, Wu J, Gao J, Liu W, Liao W (2019). MicroRNA-181c suppresses growth and metastasis of hepatocellular carcinoma by modulating NCAPG. Cancer Manag Res.

[8] Xiao C, Gong J, Jie Y, Cao J, Chen Z, Li R (2020). NCAPG Is a Promising Therapeutic Target Across Different Tumor Types. Front Pharmacol.

[9] Hu X, Xing Y, Fu X, Yang Q, Ren L, Wang Y (2020). NCAPG Dynamically coordinates the myogenesis of fetal bovine tissue by adjusting chromatin accessibility. Int J Mol Sci.

[10] Wei W, Lv Y, Gan Z, Zhang Y, Han X, Xu Z (2019). Identification of key genes involved in the metastasis of clear cell renal cell carcinoma. Oncol Lett.

[11] Liang ML, Hsieh TH, Ng KH, Tsai YN, Tsai CF, Chao ME (2016). Downregulation of miR-137 and miR-6500-3p promotes cell proliferation in pediatric high-grade gliomas. Oncotarget.

[12] Song B, Du J, Song DF, Ren JC, Feng Y (2018). Dysregulation of NCAPG, KNL1, miR-148a-3p, miR-193b-3p, and miR-1179 may contribute to the progression of gastric cancer. Biol Res.

[13] Yasui H, Kajiyama H, Tamauchi S, Suzuki S, Peng Y, Yoshikawa N (2020). CCL2 secreted from cancer-associated mesothelial cells promotes peritoneal metastasis of ovarian cancer cells through the P38-MAPK pathway. Clin Exp Metastasis.

[14] Li Z, Tang X, Luo Y, Chen B, Zhou C, Wu X (2019). NK007 helps in mitigating paclitaxel resistance through p38MAPK activation and HK2 degradation in ovarian cancer. J Cell Physiol.

[15] Grossi V, Peserico A, Tezil T, Simone C (2014). p38α MAPK pathway: a key factor in colorectal cancer therapy and chemoresistance. World J Gastroenterol.

[16] Bulavin DV, Demidov ON, Saito S, Kauraniemi P, Phillips C, Amundson SA (2002). Amplification of PPM1D in human tumors abrogates p53 tumor-suppressor activity. Nat Genet.

[17] Hrstka R, Bouchalova P, Michalova E, Matoulkova E, Muller P, Coates PJ (2016). AGR2 oncoprotein inhibits p38 MAPK and p53 activation through a DUSP10-mediated regulatory pathway. Mol Oncol.

[18] Feng Y, Liu F, Du Z, Zhao D, Cheng J, Guo W (2017). Wip1 regulates SKOV3 cell apoptosis through the p38 MAPK signaling pathway. Mol Med Rep.

[19] Chen MJ, Chou CH, Shun CT, Mao TL, Wen WF, Chen CD (2017). Iron suppresses ovarian granulosa cell proliferation and arrests cell cycle through regulating p38 mitogen-activated protein kinase/p53/p21 pathway. Biol Reprod.

[20] Tang Z, Li C, Kang B, Gao G, Li C, Zhang Z (2017). GEPIA: a web server for cancer and normal gene expression profiling and interactive analyses. Nucleic Acids Res.

[21] Gyorffy B, Lánczky A, Szállási Z (2012). Implementing an online tool for genome-wide validation of survival-associated biomarkers in ovarian-cancer using microarray data from 1287 patients. Endocr Relat Cancer.

[22] Jäger D, Stockert E, Jäger E, Güre AO, Scanlan MJ, Knuth A (2000). Serological cloning of a melanocyte rab guanosine 5'-triphosphate-binding protein and a chromosome condensation protein from a melanoma complementary DNA library. Cancer Res.

[23] Altmann S, Murani E, Schwerin M, Metges CC, Wimmers K, Ponsuksili S (2012). Maternal dietary protein restriction and excess affects offspring gene expression and methylation of non-SMC subunits of condensin I in liver and skeletal muscle. Epigenetics.

[24] Gong C, Ai J, Fan Y, Gao J, Liu W, Feng Q (2019). NCAPG Promotes The Proliferation Of Hepatocellular Carcinoma Through PI3K/AKT Signaling. Onco Targets Ther.

[25] Zhang H, Zou J, Yin Y, Zhang B, Hu Y, Wang J (2019). Bioinformatic analysis identifies potentially key differentially expressed genes in oncogenesis and progression of clear cell renal cell carcinoma. PeerJ.

[26] Chen J, Qian X, He Y, Han X, Pan Y (2019). Novel key genes in triple-negative breast cancer identified by weighted gene co-expression network analysis. J Cell Biochem.

[27] Arai T, Okato A, Yamada Y, Sugawara S, Kurozumi A, Kojima S (2018). Regulation of NCAPG by miR-99a-3p (passenger strand) inhibits cancer cell aggressiveness and is involved in CRPC. Cancer Med.

[28] Xia Z, Dickens M, Raingeaud J, Davis RJ, Greenberg ME (1995). Opposing effects of ERK and JNK-p38 MAP kinases on apoptosis. Science.

[29] Yeh HT, Tsai YS, Chen MS, Li YZ, Lin WC, Lee YR (2019). Flavopereirine induces cell cycle arrest and apoptosis via the AKT/p38 MAPK/ERK1/2 signaling pathway in human breast cancer cells. Eur J Pharmacol.

[30] Cheng MJ, Cao YG (2017). TMPYP4 exerted antitumor effects in human cervical cancer cells through activation of p38 mitogen-activated protein kinase. Biol Res.

[31] Zhao HG, Zhou SL, Lin YY, Dai HF, Huang FY (2018). Toxicarioside N induces apoptosis in human gastric cancer SGC-7901 cell by activating the p38MAPK pathway. Arch Pharm Res.

[32] Lin RC, Yang SF, Chiou HL, Hsieh SC, Wen SH, Lu KH (2019). Licochalcone A-Induced Apoptosis Through the Activation of p38MAPK Pathway Mediated Mitochondrial Pathways of Apoptosis in Human Osteosarcoma Cells In Vitro and In Vivo. Cells.

[33] Zhang B, Wu T, Wang Z, Zhang Y, Wang J, Yang B (2015). p38MAPK activation mediates tumor necrosis factor-α-induced apoptosis in glioma cells. Mol Med Rep.

[34] Cheok CF, Verma CS, Baselga J, Lane DP (2011). Translating p53 into the clinic. Nat Rev Clin Oncol.

[35] Haupt S, Berger M, Goldberg Z, Haupt Y (2003). Apoptosis - the p53 network. J Cell Sci.

[36] Lee KS, Kim SW, Lee HS (2018). Orostachys japonicus induce p53-dependent cell cycle arrest through the MAPK signaling pathway in OVCAR-3 human ovarian cancer cells. Food Sci Nutr.

